# Involvement of Phenolic Acids in Short-Term Adaptation to Salinity Stress is Species-Specific among Brassicaceae

**DOI:** 10.3390/plants8060155

**Published:** 2019-06-06

**Authors:** Ida Linić, Dunja Šamec, Jiří Grúz, Valerija Vujčić Bok, Miroslav Strnad, Branka Salopek-Sondi

**Affiliations:** 1 Department of Molecular Biology, Ruđer Bošković Institute, Bijenička c. 54, 10000 Zagreb, Croatia; ida.linic@irb.hr (I.L.); dsamec@irb.hr (D.Š.); 2 Laboratory of Growth Regulators, Institute of Experimental Botany AS CR & Faculty of Science of the Palacký University, Šlechtitelů 27, CZ-78371 Olomouc, Czech Republic; jiri.gruz@gmail.com (J.G.); miroslav.strnad@upol.cz (M.S.); 3 Department of Botany, Faculty of Science, Rooseveltov trg 6, 10000 Zagreb, Croatia; vvujcic@biol.pmf.hr

**Keywords:** Brassica crops, carotenoids, glucosinolates, polyphenols, salinity stress, seedlings, phenolic acids, tolerance

## Abstract

Salinity is a major abiotic stress negatively affecting plant growth and consequently crop production. The effects of short-term salt stress were evaluated on seedlings of three globally important Brassica crops—Chinese cabbage (*Brassica rapa* ssp. *pekinensis*), white cabbage (*Brassica oleracea* var. *capitata*), and kale (*Brassica oleracea* var. *acephala*)—with particular focus on phenolic acids. The physiological and biochemical stress parameters in the seedlings and the levels of three main groups of metabolites (total glucosinolates, carotenoids, and phenolics) and individual phenolic acids were determined. The salt treatments caused a dose-dependent reduction in root growth and biomass and an increase in stress parameters (Na^+^/K^+^ ratio, reactive oxygen species (ROS) and glutathione (GSH)) in all seedlings but most prominently in Chinese cabbage. Based on PCA, specific metabolites grouped close to the more tolerant species, white cabbage and kale. The highest levels of phenolic acids, particularly hydroxycinnamic acids, were determined in the more tolerant kale and white cabbage. A reduction in caffeic, salicylic, and 4-coumaric acid was found in Chinese cabbage and kale, and an increase in ferulic acid levels was found in kale upon salinity treatments. Phenolic acids are species-specific among Brassicaceae, and some may participate in stress tolerance. Salt-tolerant varieties have higher levels of some phenolic acids and suffer less from metabolic stress disorders under salinity stress.

## 1. Introduction

Soil salinity is an increasing problem in many areas worldwide, particularly in the semi-arid and arid Mediterranean [1,2]. Over 7% of the world’s total land and approximately 20% of irrigated land is affected by high salinity. As the extent of global soil salinization and drought events are expected to increase as a result of the global climate change [3], systematic research on salinity and drought stress tolerance mechanisms in plants and breeding tolerant crops is paramount for future food security. Salinity induces alterations in the growth and development of plants due to its cumulative effect on several physiological as well as biochemical processes such as water balance, mineral ion homeostasis, osmolyte accumulation, antioxidant metabolism, photosynthetic capacity of plants, etc. [4]. All these changes ultimately lead to huge economic losses in crop production.

*Brassica* vegetables (Brassicaceae) include many economically important species grown worldwide. These vegetables have received public and scientific attention for their health potential due to their wealth of “healthy phytochemicals” (carotenoids, phenolics, glucosinolates) [5,6,7,8,9]. They are recognized as a functional food since different epidemiological and meta-analyses have shown that metabolites found in Brassicas have anti-inflammatory, anti-oxidant, anti-mutagenic, and anti-carcinogenic activities [10,11,12].

As *Brassica* crops are commonly grown in the Mediterranean area, their production is greatly affected by unfavorable environmental conditions (abiotic stresses including increased salinity). Therefore, current pressing questions are, for example, how increased salinity affects the growth of commercially important *Brassica* crops and which of the metabolites in their tissues correlate with the tolerance to salinity of these species. These natural substances are the products of plant interaction with the environment, have limited occurrence, and some are reported to mediate abiotic stress tolerance. Their role is usually associated with protection against oxidative damage induced by abiotic stresses. However, their ecological value in plant adaption to the environment has been relatively poorly investigated. Glucosinolates, a class of specialized metabolites, almost exclusively found in Brassicaceae, have been shown to increase in plants when salinity is higher than the tolerance levels, while their production is inhibited under severe stress conditions that depend on plant species/variety [13]. Carotenoids and polyphenolic compounds are widely abundant in many plant species including Brassicaceae. Bose et al. summarized the current knowledge of the role of carotenoids and polyphenolic compounds in salinity tolerance using halophytes, a salinity-tolerant species, as a plant model for understanding complex salinity tolerance mechanisms [14]. The authors reported that halophytes have a higher concentration of carotenoids than glycophytes (a salinity-sensitive species) under control conditions and showed a much lower reduction in carotenoid concentration after salt treatment. In addition, halophytes, such as *Cakile maritima*, (Brassicaceae) accumulate polyphenolic compounds that participate in salinity tolerance due to reactive oxygen species (ROS) scavenging ability [15].

A group of phenolic compounds that may participate in abiotic stress responses are phenolic acids. Phenolic acids, including hydroxybenzoic and hydroxycinnamic acids, and their derivatives may be present in soluble forms in which they are conjugated with sugars or organic acids, as well as bound to more complex structures such as hydrolysable tannins or lignins [16]. There are reports of beneficial effects of the exogenous application of some phenolic acids to salinity-stressed plants. Miura and Tada reported that salicylic acid (SA) has great agronomic potential to improve the stress tolerance of various agriculturally important crops [17]. However, the applicability of SA is dependent on the concentration used, the mode of application, the plant species, and stage of growth. SA is a phenolic acid that acts as a stress hormone, mediating plant responses to biotic and abiotic stresses. In addition to SA, increased salinity tolerance of wheat seedlings was also obtained after treatment with sinapic, caffeic, ferulic, and p-coumaric acids [18]. Further, endogenous ferulic and p-coumaric acid are purported to be involved in the tolerant mechanism against salinity stress in rice [19]. Soil salinity also increased the concentrations of leaf phenolics, including chlorogenic acid, in honeysuckle as a mechanism for acclimation to saline stress [20]. Martinez et al. reported that cinnamic and p-coumaric acid and p-coumaryl-CoA, in addition to flavonols, were several times higher in tomato due to salinity, heat, and combined stress (heat + salinity) compared to control plants [21]. The role of phenolic acids in salinity tolerance is therefore still unclear, especially in *Brassica* crops, and needs further investigation.

Our recent paper, based on the comparative analysis of three *Brassica* crops with global economic importance, i.e., Chinese cabbage (*Brassica rapa* ssp. *pekinensis*), white cabbage (*Brassica oleracea* var. *capitata*), and kale (*Brassica oleracea* var. *acephala*) in relation to sensitivity/tolerance to salinity, identified Chinese cabbage as sensitive, white cabbage as mildly tolerant, and kale as the most tolerant species [22]. We have also shown that plant hormones play an important role in mediating salinity tolerance in the above *Brassica* crops [22,23]. In this article, we have extended our research and evaluated the effect of salinity on the levels of specialized metabolites in the same *Brassica* species (*B. rapa* spp. *pekinensis*, *B. oleracea* var. *capitata*, and *B. oleracea* var. *acephala*). We analyzed three groups of metabolites (carotenoids, glucosinolates, and phenolics) in three *Brassica* seedlings at increased salt concentrations (0–200 mM NaCl). More detailed analysis of phenolic acids was then carried out by UPLC–MS/MS. Our hypothesis was that differently tolerant *Brassica* species would respond diversely to salinity stress in terms of profile and phenolic acid levels. Correlation studies between *Brassica* crops with different tolerance to salinity stress and levels of specialized metabolites are also discussed, with a particular focus on phenolic acids.

## 2. Results

### 2.1. Physiological and Biochemical Response of Brassicas to Salinity Stress

**Figure 1:**
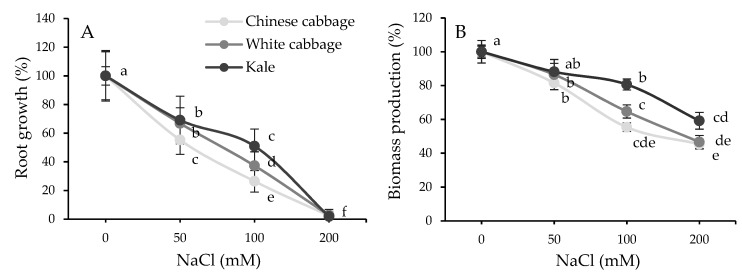
Root growth (**A**) and biomass production (**B**) of Brassica seedlings under short-term (24 h) salinity stress (0–200 mM NaCl). Points labelled with different letters differ significantly at *p* < 0.05. Data are average ± SD, *n* = 30.

The physiological effect of salt treatments was determined in planta by the root growth assay and biomass production measurements. As can be seen in Figure 1, seedling treatments with increasing NaCl concentrations caused a gradual inhibition of root growth (Figure 1A) as well as biomass production (Figure 1B) in all three Brassicas in a dose-dependent manner. The most significant reduction in biomass and root growth inhibition was observed in Chinese cabbage (*B. rapa*), then in white cabbage (*B. oleracea* var. *capitata*), and finally in kale (*B. oleracea* var. *acephala*). Thus, Chinese cabbage proved to be the most sensitive species, whereas kale was the most tolerant under our experimental conditions.

**Figure 2:**
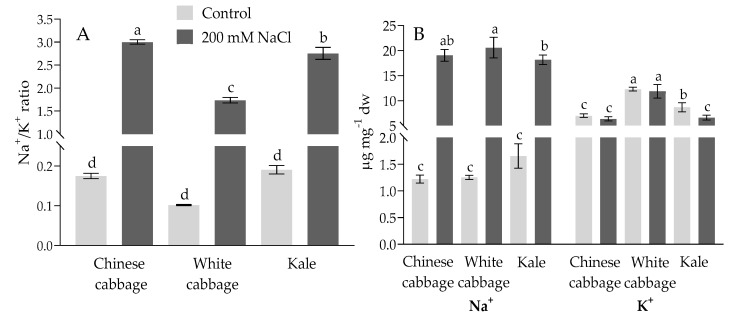
Brassica seedlings’ NA^+^/K^+^ ratio (**A**), and Na^+^ and K^+^ content (**B**) under control and salt stress (200 mM NaCl) treatments. Data are averages ± SD (*n* = 4). Points labelled with different letters differ significantly at *p* < 0.05.

Na^+^ and K^+^ ion levels in Brassica seedlings are presented in Figure 2. As shown, white cabbage had the highest basal level of potassium (12.31 µg mg^−1^ dry weight (dw)), followed by kale (8.67 µg mg^−1^ dw) and Chinese cabbage (6.98 µg mg^−1^ dw), while sodium concentrations were similar in all three types of seedlings (between 1.2 and 1.6 µg mg^−1^ dw). Salt treatment (200 mM NaCl) did not significantly change the K^+^ level in white cabbage, though there was a drop in Chinese cabbage and kale in comparison to the control (Figure 2B). The Na^+^/K^+^ ratio increased in all three varieties on salinity, mainly due to increased Na^+^ concentration in seedlings (Figure 2A). The most marked increase in the Na^+^/K^+^ ratio was in Chinese and white cabbage (17.2- and 17.1-fold) and then kale (14.5-fold) compared to the controls.

**Figure 3:**
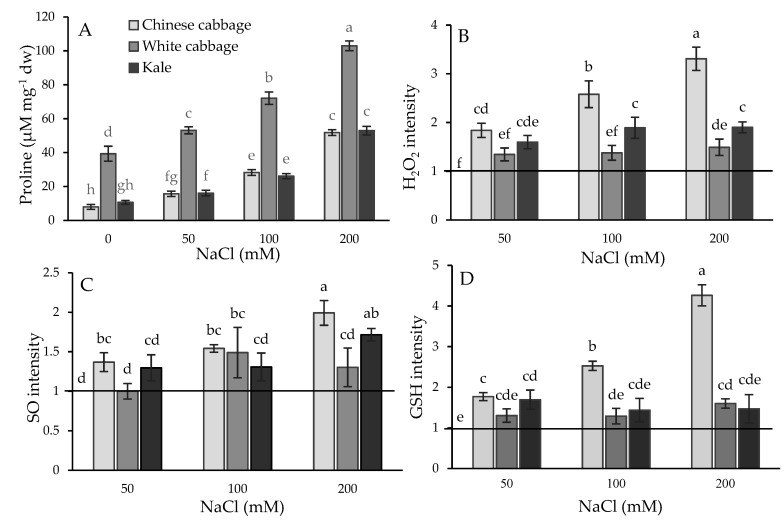
Biochemical stress markers: proline content (**A**) and fluorescence intensity due to the reactive oxygen species (ROS) H_2_O_2_ (**B**), and superoxide (SO) (**C**), as well as glutathione (GSH) (**D**) in the roots of Chinese cabbage, white cabbage, and kale seedlings under increasing salt concentrations (0, 50, 100, and 200 mM NaCl). Data in figures B, C, and D were normalized according to the corresponding controls (horizontal lines normalized to 1). Data are averages ± SD (*n* = 5). Points labeled with different letters differ significantly at *p* < 0.05.

In order to check the stress status of the treated seedlings, we measured the proline content and the ROS and glutathione (GSH) levels as biochemical stress markers (Figure 3). Interestingly, white cabbage had a significantly higher basic proline level (in control samples), 5- and 3.6-fold higher than Chinese cabbage and kale, respectively. In salt treatments, the proline levels increased significantly in all three Brassica seedlings in a dose-dependent manner. The most pronounced increase was in white cabbage (6.65-fold) at 200 mM NaCl compared to the control.

ROS levels (superoxide and H_2_O_2_) were also elevated in all Brassica seedlings (Figure 3). Following salt treatment, the most prominent increase in SO and H_2_O_2_, was obtained in Chinese cabbage in a dose-dependent manner (reaching 2-fold and 3.3-fold at 200 mM NaCl, respectively, compared to the control), although this trend was not noticed in white cabbage and kale. SO was higher, about 1.5 and 1.7-fold, while H_2_O_2_ was increased 1.5 and 1.9-fold in white cabbage and kale at higher salt concentrations compared to the corresponding controls.

GSH levels were also elevated under salt stress conditions, most notably in Chinese cabbage in a dose-dependent manner, up to 4.3-fold at 200 mM NaCl compared to the control. Its level in kale was significantly increased up to 1.7-fold (at 50 mM NaCl) and did not show a tendency to further increase at higher salt concentrations, while in white cabbage it was significantly increased in severe salt conditions (200 mM NaCl), up to 1.6-times compared to the controls.

### 2.2. Pigment Changes in Brassica Seedlings under Salinity Stress

Control kale and white cabbage had higher chlorophyll (Chl) content than Chinese cabbage (0.89 mg g^−1^ fresh weight (fw) in kale, 0.74 mg g^−1^ fw in white cabbage, and 0.59 mg g^−1^ fw in Chinese cabbage) (Table 1). The Chl *a* changed at 100 mM NaCl in Chinese and white cabbage. There was no change in kale in the salt conditions. The Chl *a*/Chl *b* ratio remained unchanged following exposure of the Brassicas to salinity (Table 1).

### 2.3. Correlations between Specific Metabolites and Brassica Species under Salinity Stress

**Figure 4:**
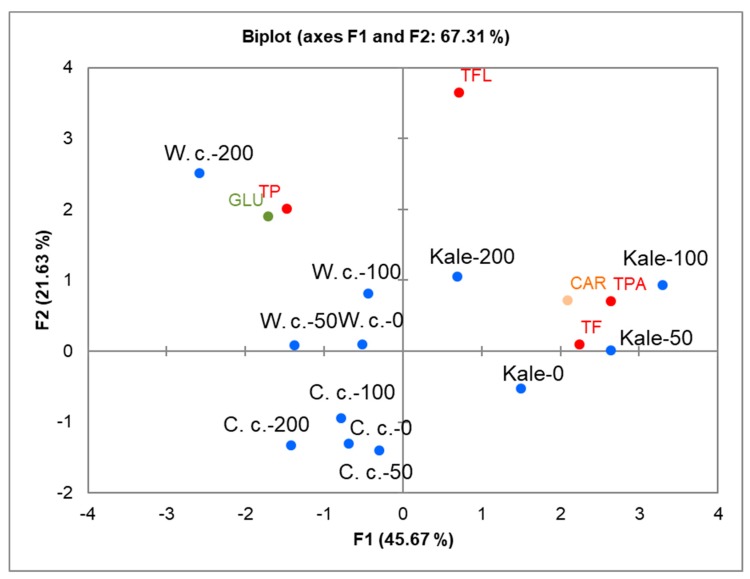
Two-dimensional principal component analysis (2D-PCA) of specialized metabolites in Chinese cabbage (C. c), white cabbage (W. c.), and kale (Kale) after 24 h exposure to 0 (control), 50, 100, and 200 mM NaCl. The green, orange, and red symbols indicate the positions in the score plots of glucosinolates (GLU), carotenoids (CAR), and phenolic compounds (total phenolics (TP), total flavonoids (TF), total phenolic acids (TPA), total flavonols (TFL)), respectively. The blue symbols indicate the positions of the Brassica crops following each of the treatments. PCA was created based on data presented in Supplementary Table S1. The correlation matrix used, eigenvalues, factor loadings, and factor scores are given in Supplementary Tables S2–S6.

Groups of selected metabolites, including total carotenoids (CAR), total glucosinolates (GLU), and total phenolic compounds (total polyphenols, TP, total phenolic acids, TPA, total flavonoids, TF, and total flavanols, TFL), were measured spectrometrically in control and treated seedlings (Supplementary Table S1). To investigate correlations among the Brassicas in terms of the measured specialized metabolites, the data were subjected to Principle Component Analysis (PCA) based on a matrix of Pearson correlation coefficients (*p* < 0.05). The positions of the Brassicas and the relations among the measured parameters under the salinity treatments are shown as PCA biplots in Figure 4. The resulting correlation matrix, eigenvalues, factor loadings, and factor scores are presented in Supplementary Tables S2–S6. The first two Principal Components, F1 and F2, explained 67.31% of the cumulative variability of the measured traits (see also Table S3). The separation of cultivars clearly showed that their response to salinity apropos specific metabolites varied considerably. Chinese cabbage appeared positioned in the lower left quadrant, white cabbage in the upper left quadrant, and kale in the upper right quadrant. The PCA plot also shows the relationships of the parameters in Brassicas salinity responses. The metabolites analyzed under stress conditions are grouped mainly near white cabbage and kale. GLU and TP are grouped near white cabbage treated with a strong salt concentration (200 mM NaCl), while CAR, TPA, and TF are placed near kale treated with mild to high salinity (50–100 mM NaCl). Pearson linear coefficients (Table S2) showed positive correlations among the parameters grouped closely; TPA positively correlated with TF (0.724) and CAR (0.652).

### 2.4. Phenolic Acid Profiles and Their Dynamic under Salinity Stress Conditions

In this case, we carried out a more detailed profiling of phenolic acids using the UPLC–MS/MS method in control and salt-treated *Brassica* seedlings. In total, 10 phenolic acids were identified on the basis of retention time (RT) and multiple reaction monitoring transition (MRM) (Table 2): caffeic acid (CaA), ferulic acid (FA), gallic acid (GaA), 4-hydroxybenzoic acid (pHBA), protocatechuic acid (PA), 4-coumaric acid (pCoA), SA, sinapic acid (SiA), syringic acid (SyA), and vanillic acid (VA). Representative chromatograms of the identified phenolic acids in kale seedlings are shown in Supplemental Figure S1.

We measured soluble (free and ester-bound phenolic acids) and insoluble cell wall-bound phenolic acids. Total phenolic acids are the result of the sum of free phenolic acids and the acids determined after hydrolysis in soluble and insoluble fractions. By comparing the controls (untreated seedlings of the three species), we found the highest level of total phenolic acids under these experimental conditions in kale (8.4 nmol mg^−1^ dw), then in white cabbage (7.3 nmol mg^−1^ dw), and finally in Chinese cabbage (6.0 nmol mg^−1^ dw).

The most abundant phenolic acid in all three Brassicas was SiA (79–84% of total phenolic acid content) (Table 2). Other phenolic acids were less abundant (in the range 0.6–6.4% of total phenolic acid content). The selected Brassica species differed significantly in the level of individual phenolic acids. Chinese cabbage had higher amounts of VA and SA compared to the other species, while white cabbage had the highest levels of FA and SyA, and kale was very rich in CaA and SiA. White cabbage and kale also had a significantly higher level of PA, GaA, CaA, and SiA compared to Chinese cabbage (Table 2).

The profile of phenolic acids, including free, ester-bound, and cell-wall bound phenolic acids in the salt-treated seedlings and corresponding controls is shown in Table 3. The general observation is that the levels of the free forms of phenolic acids were low in overall phenolic acid content, suggesting that phenolic acids are abundantly conjugated as glycosides and bound on the cell wall in Brassicas. Indeed, some phenolic acids (GaA, VA, CaA, and SyA) were not identified in free form at all. Only three phenolic acids, pCoA, SiA, and FA, were detected as cell wall-bound. White cabbage contained the highest level of cell wall-bound phenolic acids compared to kale and Chinese cabbage.

The most pronounced saline-based phenolic acid deficiency was observed in Chinese cabbage compared to the corresponding control. Thus, the levels of free PA, pCoA, and SA were significantly reduced in the most severe stress conditions (200 mM NaCl), while CaA was decreased in all salinity treatments compared to the control. The metabolism of phenolic acids did not change markedly under salinity in white cabbage. In kale, we observed a significant decrease in CaA (total) and SA (free form) at 100 and 200 mM NaCl, a decrease of pCoA at all salt concentration, and a significant increase in free FA level at the strongest salt concentration.

**Figure 5:**
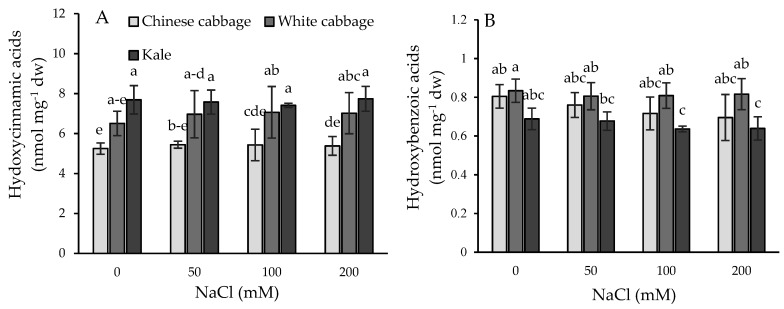
Total hydroxycinnamic acids (**A**) and total hydroxybenzoic acids (**B**) in Brassica seedlings upon salinity treatments (0–200 mM). Data are averages ± SD (*n* = 5). Points labeled with different letters differ significantly at *p* < 0.05.

Total hydroxycinnamic acids (including pCoA, CaA, FA, and SiA) versus total hydroxybenzoic acids (including PA, GaA, VA, pHBA, SyA, and SA) in the treated Brassica seedlings are shown in Figure 5. Kale had the highest level of total hydroxycinnamic acids, followed by white and then Chinese cabbage. In contrast, Chinese and white cabbage had a higher level of total hydroxybenzoic acid than kale. There was a trend of a mild but not statistically significant increase in total hydroxycinnamic acids and a mild decrease in total hydroxybenzoic acids in all three species. In the group of hydoxybenzoic acids, a significant decrease was found for SA in Chinese cabbage and kale upon salinity treatment.

## 3. Discussion

### 3.1. Stress Status of Seedlings: Physiological and Biochemical Parameters

Salinity is a complex abiotic stress generating three unfavorable conditions for plant growth and development: ionic stress caused by toxic concentrations of ions (mainly Na^+^), osmotic stress due to associated reduction in water uptake, and oxidative stress mainly driven by increased levels of ROS [24]. The results presented here showed the negative effects of short-term salinity stress on biomass production and root growth of seedling of three *Brassica* species (Chinese cabbage, white cabbage, and kale). The most pronounced inhibition of root growth and biomass production was observed in Chinese cabbage. This is in line with our recent data from four-week-old *Brassica* plants exposed to salinity, which showed that kale is the most tolerant of the three species, while Chinese cabbage is the most sensitive to salt stress [22]. In parallel, the Na^+^/K^+^ ratio, a salinity stress marker, increased significantly under salinity stress in all three species, but a greater increase was observed in Chinese and white cabbage compared to kale. Maintaining a low tissue Na^+^/K^+^ ratio has been suggested as an important selection criterion for salt tolerance in Brassicas [25,26].

As mentioned, increased ROS production in plants is usually associated with stress conditions. Here, we showed that salinity stress increases ROS production (SO and H_2_O_2_) in seedling roots in a dose-dependent manner in all three *Brassica* seedlings studied but especially in Chinese cabbage. ROS are formed in all aerobic organisms due to multistage oxygen reduction. The first step in oxygen reduction is the formation of superoxide (O2^−^) which is rapidly converted to hydrogen peroxide (H_2_O_2_) by superoxide dismutases. H_2_O_2_ is the most stable of the ROS.

In addition, the osmoprotective proline was increased in a dose-dependent manner in all varieties. Proline is recognized as a multi-functional molecule that accumulates in response to various abiotic stresses. It acts as an osmoprotectant and as a redox-buffering agent that has antioxidant properties under stress conditions [27]. Note that white cabbage contained a significantly higher basic proline level (control seedlings) than other varieties and reached the highest proline level after salinity treatments. Ashraf and McNeilly [25] reported that salt-tolerant *Brassica* cultivars accumulate significantly higher levels of proline in the leaves compared to salt-sensitive cultivars, which is consistent with our data. The beneficial effect of proline hyperaccumulation on salt tolerance has been demonstrated in *Thellungiella salsuginea* and *Lepidium crassifolium*, two halophytic wild relatives of *Arabidopsis* that accumulate more proline under control and salt-stressed conditions [14].

Another non-enzymatic compound regulating ROS levels and an informative marker of abiotic stress is GSH. GSH levels were increased under salinity stress in all three Brassicas in a dose-dependent manner. In a previous report, it was shown that halophytic *Lycopersicon pennellii* had an increased content of reduced glutathione compared to its glycophyte relative *Lycopersicon esculentum* [14].

### 3.2. Selected Metabolites Responsive to Salinity Stress

Some of the multiple biochemical pathways involved in plant defense against salinity stress lead to the synthesis of selected metabolites [28]. We evaluated several groups of natural substances, including total CAR, total GLU, and TP compounds (TPA, TF, and TFL) under increased salinity stress.

Kale and white cabbage contained higher CAR levels than Chinese cabbage, although the difference was not statistically significant under our experimental conditions. Short-term salinity stress did not significantly affect CAR content in any of the investigated species. Published data show that the salt-tolerant monocotyledonous *Chloris virgate* contains a constitutively higher carotenoid concentration under control conditions than the more sensitive wheat and shows a much smaller reduction in carotenoid concentration upon exposure to increased salt concentrations [29].

The GLU content was similar in untreated Chinese cabbage, white cabbage, and kale and was significantly increased in salinity treatments for all varieties, but the highest increase was observed in white cabbage. Changes in the amount and pattern of total glucosinolates in plants were found to be related to developmental stage, plant species, and duration of salinity stress. López-Berenguer et al. [30] showed that low salinity (40 mM) greatly increased the total GLU content of broccoli inflorescence, while at higher salinity (80 mM), the GLU content was reduced. Further, the GLU contents of 5- and 7-day-old radish sprouts were significantly increased, and the myrosinase activities were inhibited by 100 mM NaCl treatment [31]. Long-term salinity stress (21 days) and exposure to chloride salts caused a decrease in aliphatic GLUs in shoots of *B. rapa* plants, corresponding to reduced transcript level of *CYP79F1* coding for the key enzyme for their biosynthesis. Similarly, elevated levels of indole and aromatic GLUs by Na_2_SO_4_ coincided with increased gene expression of enzymes responsible for the biosynthesis of these GLUs [32].

As far as polyphenolic compounds are concerned, all Brassicas contained similar TP contents, which did not change significantly under salinity stress. However, some groups of polyphenols differed in these three species and varied in different salinity conditions. Thus, kale seedlings contained the highest level of TPA than other varieties, while white cabbage and kale contained significantly higher levels of TFL than Chinese cabbage. As a result of salinity, a significant increase in TPA and TFL were observed only in kale, while TF was significantly increased in Chinese cabbage. Published data showed that the TP content was twice as high for salt-tolerant species such as *Tamarix gallica*, *Limoniastrum monopetalum*, *Limoniastrum guyonianum*, *Suaeda fruticosa*, and *Mesembryanthemum edule* compared to salt-sensitive species (*Mentha pulegium* and *Nigella sativa*) [33,34]. The TP content of 3- and 5-day-old sprouts of *Raphanus sativus* was significantly increased by 100 mM NaCl treatment [31].

### 3.3. Phenolic Acids in Plant Stress Responses

Although each type of stress has its own characteristics, almost all abiotic stressors change the cellular redox state, and therefore the participation of antioxidants in the plant stress responses is essential. Phenolic acids, due to their structure, are known as strong antioxidants that can contribute to ROS scavenging [35]. Hydroxycinnamic acids exhibit higher antioxidant activity than similar hydroxybenzoic acids, since their CH=CH–COOH side chain is considered to possess larger hydrogen donor and radical stabilizing properties [36].

We found that untreated kale seedlings contained a higher content of total phenolic acids than white and Chinese cabbage (Table 2). These phenolic acids are mostly present in conjugated forms, consistent with previously published data [5]. In addition, kale had the highest levels of hydroxycinnamic acids but the lowest content of hydroxybenzoic acids compared to the other two species (Figure 5). The ratio of total hydroxycinamic to total hydroxybenzoic acids was 11.2 in the control kale seedlings, 7.8 in white cabbage, and 6.5 in Chinese cabbage. After salinity treatments, this ratio constantly increased to 12.1, 8.6, and 7.7 in kale, white, and Chinese cabbage at 200 mM NaCl, respectively. It follows that short-term exposure of plants to increased salinity can cause the accumulation of total hydroxycinnamic acids over hydroxybenzoic acids in Brassicas. An increase in hydroxycinnamic acid content (especially 1,3-dicaffeoylquinic acid, 1-feruoyl-5-caffeoylquinic acid, and 3-caffeoyl-1-5-quinolactone) has been described in tomato plants exposed to salinity and combined heat and salinity [21]. These authors found a more pronounced increase in hydroxycinnamic acids than we demonstrated in our experiments. This may be due to the duration of stress and the growth stage of the plants. In their experiments, tomato plants were exposed to long-term stress for 21 days compared to our 24 h salt-stressed seedlings. A significant increase of p-cumaric and caffeic acids was reported in cabbage after osmotic dehydration [37]. Furthermore, it has been found that hydroxycinnamic acids (chlorogenic acid, caffeic acid, 1,3-dicaffeoylquinic, and 1,5-dicaffeoylquinic acid) were increased in artichoke plants grown under water deficit [38].

The most abundant phenolic acid in all three *Brassica* seedlings was SiA, which has already been shown to be dominant in a range of *Brassica* crops [5,11,39,40,41]. The higher salinity tolerance under our experimental conditions for kale and white cabbage compared to Chinese cabbage was probably associated with higher levels of hydroxicinnamic acids SiA, CaA, and FA and of hydroxybenzoic acids PA, GA, 4-HBA, and SyA. Furthermore, white cabbage and kale contain higher levels of cell wall-bound phenolic acids (SiA, FA, and pCoA) than Chinese cabbage. The published data show that FA and pCoA are involved in tolerance mechanisms for salinity stress in rice [19]. Gupta and De [42] showed that salt-tolerant rice varieties increase FA and pCoA as key components bound to cell wall formation under salt stress. CaA is elevated under salinity in some *Echinacea sp.* [43]. The exogenous application of phenolic acids can reduce electrolyte leakage in NaCl-treated wheat seedlings, where maximum decrease was observed in the presence of sinapic acid followed by caffeic, ferulic, and p-coumaric acids [18]. Further, lipid peroxidation decreased or remained unaffected, while H_2_O_2_ levels dropped at the maximum in seedlings treated with caffeic acid. The scavenging capacity of hydroxyl radicals in seedlings grown under strong salt stress increased to the maximum after caffeic and sinapic acid treatments. Hydroxycinnamic acids are very effective antioxidants depending on their structure [44,45]. The proven sequence of efficacy was as follows: caffeic > sinapic > chlorogenic > ferulic > p-coumaric acid [46].

Among hydroxybenzoic acids, SA is a well-known signaling molecule involved in abiotic stress responses. The exogenous use of salicylic acid and its derivatives has been reported to alleviate oxidative stress arising under salinity conditions [47]. Here, we found that Chinese cabbage contained the highest level of SA under control conditions compared to white cabbage and kale. Under the applied salt-stress conditions, a significant decrease in SA content was observed in Chinese cabbage (2.8-fold) and kale (1.4-fold) under severe stress (200 mM NaCl), while the SA content was not significantly affected in white cabbage (the reduction was 1.3-fold at 200 mM NaCl). More tolerant Brassicas are capable of maintaining unchanged SA levels, or their SA level is reduced less than that of sensitive species. The correlations between endogenous SA levels and salinity tolerance are controversial. Research with mutants with altered endogenous SA concentrations showed no clear pattern of behavior during salt stress. Some studies have found that SA-deficient Arabidopsis *NahG* plants showed increased growth compared to wild-type plants and SA-hyperaccumulation (*snc1*) mutants during salinity stress. However, in other studies, SA-hyperaccumulation mutants, namely, *siz1* (a small ubiquitin-like modifier E3 ligase1) showed increased growth, while a significant growth inhibition was observed in SA-deficient plants (*NahG*, *sid2*, and *eds5*) during salt stress [48].

## 4. Materials and Methods

### 4.1. Plant Material and Experimental Conditions

Chinese cabbage (*B. rapa* var. *pekinensis*) was obtained from International Seeds Processing GmbH, Germany, while white cabbage (*B. oleracea* var. *capitata* cv. Varaždinski) was obtained from the Agricultural Advisory Service of Varaždin Region, Croatia, and kale seeds (*B. oleracea* var. *acephala*) from a family farm from Vrgorac, Croatia. Before germination, the seeds were surface-sterilized in 3% Izosan G (Pliva, Croatia) for 10 min, washed with sterile water (5 times), transferred to 1% agar plates, and left at +4 °C for three days in the dark. The seeded plates were placed in a growing chamber, in a vertical position, under control conditions of 16/8 h light/dark photoperiod, light intensity of 115 µmol m^−2^ s^−1^, and temperature 22 °C. After the seedlings reached about 1 cm in length, they were placed on 1% agar plates containing NaCl (in concentration range 50–200 mM). Corresponding controls were placed on 1% agar without salt. Both control and experimental plates were incubated for 24 h. Biomass, root growth, and ROS production were determined in in vivo seedlings. For biochemical analysis, five biological replicates of seedlings were collected, immediately frozen using liquid nitrogen, and stored at −80 °C. The plant material was then freeze-dried and stored until analysis.

### 4.2. Determination of Sodium and Potassium Content

Levels of Na^+^ and K^+^ in *Brassica* seedlings were determined by high-resolution inductively coupled plasma mass spectrometry (HR-ICP–MS, Element 2, Thermo, Bremen, Germany) in connection with an autosampler ESI-a SC-2 DX FAST (Elemental Scientific, Omaha, NE, USA). The measurement parameters and instrument conditions were set as described earlier [49]. Indium was used as an internal standard. Lyophilized seedling tissues (about 100 mg) were subjected to microwave- (Anton Paar Multiwave 3000, Graz, Austria) assisted acidic digestion in HNO_3_/HF (60:1, *v*/*v*) at 1400 W. The measurements were performed in four replicates.

### 4.3. Proline Quantification

Proline concentrations were assayed according to [50] with some modifications. In brief, extraction was performed using 30 mg of the freeze-dried tissue in 70% ethanol. A volume of 100 µL of the extract was mixed with 1000 µL of the reaction mixture (1% ninhydrin [*w*/*v*], 60% acetic acid [*v*/*v*], and 20% ethanol [*v*/*v*]) and then heated to 95 °C for 20 min. Proline levels were measured at 520 nm using a UV–VIS spectrophotometer (BioSpec-1601 E, Shimadzu) and calculated using a standard curve (y = 0.0015x, R^2^ = 0.9991; serial concentrations of proline standard (Sigma): 0.04, 0.1, 0.2, 0.4, 1.0, 1.5 mM). The results are expressed in µM L-proline mg^−1^ dw (dry weight).

### 4.4. ROS and GSH Fluorescent Measurements

The amount of ROS (SO, H_2_O_2_) as well the GSH content of the seedlings was determined in vivo using specific dyes (dihydroethidium, DHE, dichlorodihydrofluorescein diacetate, DCFH-DA, and monochlorobimane, MCB) according to reported methods [51]. All measurements were performed with the roots of stressed seedlings, compared to their appropriate controls. Briefly, the roots of stress and control seedlings were incubated in 10 μM DHE (30 min), 50 μM DCFH-DA (30 min), and 50 μM MCB (40 min) for the determination of SO, H_2_O_2_, and GSH, respectively. After incubation, the samples were washed with water to remove the dye surplus. Fluorescent signals that appeared as a result of the reaction between fluorescent dyes and substrates were determined using a fluorescent microscope (Olympus BX51, Olympus Optical Co. (Europa) GmbH) connected to the camera (Olympus DP70, Tokyo, Japan). The accumulated fluorescent products were quantified using Lucida 6.0 software (Kinetic Imaging Ltd., Wirral, UK). Twenty-five fields on each image were analyzed, and the results are presented as the mean of the fluorescence intensity of five images per treatment.

### 4.5. Pigment Content Determination

Plant pigments, chlorophylls *a* and *b*, and carotenoids were measured in fresh cotyledons of seedlings upon treatments, and their contents were calculated according to Lichtenthaler and Buschmann [52]. Pigments levels were measured at three different wavelengths, 663.2 nm for chlorophyll *a*, 646.8 nm for chlorophyll *b*, and 470 nm for carotenoids. The results are presented in mg g^−1^ fw (fresh weight).

### 4.6. Glucosinolate Measurements

Total glucosinolate content was measured according to Aghajanzadeh et al. [53] with certain adjustments. Lyophilized tissue (30 mg) was extracted in 80% methanol. In order to inactivate the myrosinase enzyme, the extracts were subsequently heated in a thermobloc at 95 °C for 2 min and then were cooled and centrifuged (5 min at 13 000 rpm). Glucosinolate levels were determined in a reaction mixture (930 µL) containing 30 µL methanolic plant extract and 900 µL 2 mM disodium tetrachloropalladate (Na_2_PdCl_4_) using a UV–VIS spectrophotometer (BioSpec-1601 E, Shimadzu) at 425 nm. The samples were incubated for 30 min at room temperature before the measurements. The results were calculated using a standard curve (y = 0.0003x, R^2^ = 0.998; serial concentrations of sinigrin standard (Carl Roth GmbH, Karlsruhe, Germany): 0.1, 0.25, 0.5, 1.0, 1.5, 3.0 mg mL^−1^) and are presented as sinigrin equivalents per dry weight (μg sinigrin mg^−1^ dw).

### 4.7. Determination of Polyphenolic Compounds

For the measurement of polyphenolic compounds, extractions were carried out in 2 mL 80% methanol using 60 mg of freeze-dried tissue. For tissue homogenization, a Mixer Mill MM 400 (Retsch, Haan, Germany) was used for 5 min at 30 Hz, after which the extracts were placed in a sonicator (10 min) and further mixed in a tube rotator (1 h, 15 rpm). The extracts were then centrifuged (Eppendorf centrifuge, 10 min, 13,000 rpm), and the supernatants were used for all analyses described below. All extractions were carried out in five biological replicates for all three species. The measurements were adapted to small volumes.

The Folin–Ciocalteu method for the assessment of TP was used according to Singleton and Rossi [54]. The results were calculated using a standard curve (y = 0.0011x, R^2^ = 0.998; serial concentrations of gallic acid (Alfa Aesar, Haverhill, MA, USA): 50, 100, 150, 250, 500 mg L^−1^) and are presented as equivalents of gallic acid per dry weight (mg GAE mg^−1^ dw). TPA were determined using Arnow’s reagent according to the European Pharmacopoeia [55]; the results were calculated by using a standard curve (y = 0.0042x, R^2^ = 0.9936; serial concentrations of caffeic acid (Sigma-Aldrich, St. Louis, MO, USA): 10, 50, 100, 250, 500 mg L^−1^) and are expressed as equivalents of caffeic acid per dry weight (mg CAE mg^−1^ dw). TF were measured using the AlCl_3_ method [56]. The results were calculated by using a standard curve (y = 0.0031x, R^2^ = 0.9898; serial concentrations of catechin standard (Kemika, Zagreb, Croatia): 50, 100, 150, 200, 250 mg L^−1^) and are presented as equivalents of catechin per dry weight (mg CE mg^−1^ dw). TFL were analyzed by the p-dimethylaminocinnamaldehyde (DMACA) method [57]. The results were calculated by using a standard curve (y = 0.1414x, R^2^ = 0.9996; serial concentrations of catechin standard (Kemika, Zagreb, Croatia): 0.5, 1, 2, 4, 6, 8, 10 mg L^−1^) and are presented as equivalents of catechin per dry weight (mg CE mg^−1^ dw).

### 4.8. Principle Component Analysis (PCA)

Relations between the measured values of specific metabolites (total phenolics, flavonoids, phenolic acids, flavanols, glucosinolates, and carotenoids) and the experimental variants of *Brassica* crops were examined by PCA. PCA was performed using a correlation matrix of the average values of traits after standardization (autoscaling). Linear correlations among variables were determined by Pearson coefficients (*p* < 0.05). The XLSTAT software (ver. 2017.01.40777) implemented in Microsoft Office Excel 2010 was used for all statistical procedures.

### 4.9. Phenolic Acid Analyses

The extraction of phenolic acids was performed using 30 mg of freeze-dried plant material in 80% methanol. Internal standards of deuterium-labeled 4-hydroxybenzoic and salicylic acids were added to all samples at a final concentration of 10^−6^ mol L^−1^. The fractions of soluble free acids, soluble ester-bound phenolic acids, and cell wall-bound phenolic acids were prepared by a previously published method [58]. Quantification and identification of phenolic acids were performed using UPLC–MS/MS as described earlier [59].

### 4.10. Statistical Analysis

The data were analyzed with the STATISTICA program (Version Stat Soft. Statistica.v 10.0. Enterprise). ANOVA was used to analyze the relevant factors, and values were considered to be significant at *p* < 0.05. Post-hoc multiple mean comparison (Tukey’s HSD test) was used for multiple comparisons.

## 5. Conclusions

In this work, we evaluated the effect of short-term (24 h) salt stress (influence of NaCl at concentrations of 50, 100, or 200 mM) on selected metabolites, with a special focus on phenolic acids, in Chinese cabbage, white cabbage, and kale seedlings. The negative effects of increased salinity were confirmed in all experimental variants. Seedlings showed root growth inhibition, reduced biomass production, and increased Na^+^/K^+^ ratio, elevated ROS and GSH, and proline content. Based on these parameters, a high level of stress was demonstrated due to increased salinity, especially in Chinese cabbage, suggesting that this Brassica is highly sensitive to salinity stress. PCA analysis showed a grouping of specific metabolites (carotenoids, polyphenols, and glucosinolates) closer to more tolerant species, suggesting a positive role for these natural substances in stress management. The phenolic acid analysis further confirmed that the more tolerant Brassicas, kale and white cabbage, contained a significantly higher level of total phenolic acids, especially of total hydroxycinnamic acids with respect to hydroxybenzoic acids, compared to Chinese cabbage. Total hydroxycinnamic acids tended to increase, while hydroxybenzoic acids tended to decrease under the applied salinity conditions. Furthermore, the more tolerant white cabbage and kale contained higher levels of cell wall-bound phenolic acids, particularly SiA, compared to the salt-sensitive Chinese cabbage. The most marked decrease in phenolic acids (especially PA, pCoA, SA, and CaA) was observed in salt-treated Chinese cabbage. White cabbage showed no significant changes in phenolic acid levels. In addition to the decrease in CaA, SA, and pCoA, there was also a significant increase in FA in kale under stress conditions. Our results suggest that phenolic acids are species-specific in Brassicaceae and can contribute to their stress tolerance. Salt-tolerant species exhibit a higher level of phenolic acids and suffer less from metabolic disorders under salinity stress.

## Figures and Tables

**Table 1:** Chlorophylls (Chl) levels (mg g^−1^ fresh weight (fw)) upon treatments with increasing concentrations of NaCl (mM). Data are averages ± SD (*n* = 5).

*Brassica* crop	NaCl (mM)	Chl *a*	Chl *b*	Total chl	Chl *a*/Chl *b*
Chinese cabbage	0	0.42 ± 0.04 ^d^	0.17 ± 0.03 ^c^	0.59 ± 0.07 ^c^	2.48 ± 0.24 ^a^
	50	0.49 ± 0.03 ^bcd^	0.19 ± 0.01 ^bc^	0.68 ± 0.04 ^de^	2.54 ± 0.09 ^a^
	100	0.64 ± 0.09 ^ab^	0.25 ± 0.04 ^abc^	0.89 ± 0.14 ^abcd^	2.60 ± 0.03 ^a^
	200	0.45 ± 0.07 ^cd^	0.22 ± 0.03 ^bc^	0.67 ± 0.06 ^de^	2.07 ± 0.52 ^ab^
White cabbage	0	0.49 ± 0.02 ^bcd^	0.25 ± 0.05 ^abc^	0.74 ± 0.07 ^bcde^	2.00 ± 0.35 ^ab^
	50	0.53 ± 0.06 ^abcd^	0.24 ± 0.04 ^abc^	0.78 ± 0.09 ^abcde^	2.20 ± 0.21 ^ab^
	100	0.68 ± 0.08 ^a^	0.33 ± 0.05 ^a^	1.00 ± 0.12 ^a^	2.09 ± 0.28 ^ab^
	200	0.49 ± 0.09 ^bcd^	0.20 ± 0.04 ^bc^	0.69 ± 0.14 ^cde^	2.48 ± 0.06 ^a^
Kale	0	0.62 ± 0.08 ^abc^	0.27 ± 0.03 ^ab^	0.89 ± 0.11 ^abcd^	2.33 ± 0.27 ^ab^
	50	0.69 ± 0.05 ^a^	0.27 ± 0.01 ^ab^	0.95 ± 0.03 ^ab^	2.57 ± 0.32 ^a^
	100	0.62 ± 0.03 ^abc^	0.24 ± 0.02 ^abc^	0.86 ± 0.05 ^abcd^	2.56 ± 0.18 ^a^
	200	0.59 ± 0.05 ^abcd^	0.34 ± 0.02 ^a^	0.92 ± 0.07 ^abc^	1.76 ± 0.07 ^b^

Points labeled with different letters (a–d) differ significantly at *p* < 0.05. Statistical analysis has been performed in each column, among three Brassica species.

**Table 2:** List of determined phenolic acids by UPLC–MS/MS, their retention times (RT) and multiple reaction monitoring transition MRM. Total levels of particular phenolic acids were expressed (pmol mg^−1^ dw) in untreated seedlings of Chinese cabbage, white cabbage, and kale. Color gradation from light yellow to dark green comparatively represents values among species from the lowest to the highest.

Compound (Abbreviation)	RT	MRM	Chinese Cabbage	White Cabbage	Kale
	min	*m*/*z*	pmol mg^−1^ dw		
protocatechuic acid (PA)	3.84	153 > 109	79.13 ± 4.17b	173.79 ± 39.78a	136.92 ± 15.84a
galic acid (GaA)	2.64	153 > 109	65.49 ± 9.26b	124.03 ± 11.81a	118.66 ± 6.65a
4-hydroxybenzoic acid (pHBA)	4.84	137 > 93	120.03 ± 29.46b	174.89 ± 16.07a	147.07 ± 8.58ab
vanillic acid (VA)	5.44	167 > 152	291.96 ± 37.29a	142.38 ± 26.87b	153.14 ± 14.22b
caffeic acid (CaA)	5.37	179 > 135	34.19 ± 5.23c	51.30 ± 2.35b	149.61 ± 9.53a
syringic acid (SyA)	5.64	197 > 182	72.60 ± 10.41b	98.61 ± 11.01a	51.13 ± 4.86c
4-coumaric acid (pCoA)	6.55	163 > 119	127.83 ± 34.47a	178.18 ± 47.41a	189.08 ± 23.25a
sinapic acid (SiA)	7.05	223 > 208	4834.88 ± 290.75c	5807.30 ± 677.05b	7073.88 ± 669.84a
ferulic acid (FA)	7.14	193 > 178	253.02 ± 14.59b	469.18 ± 95.30a	275.67 ± 29.17b
salicylic acid (SA)	8.25	137 > 93	175.62 ± 46.53a	120.49 ± 24.20b	81.78 ± 4.17b
Total			6054.78 ± 249.04c	7340.17 ± 621.87b	8376.94 ± 707.89a

Data labeled with different letters (a–c) differ significantly at *p* < 0.05 for each particular phenolic acid among the three species. Statistical analysis has been performed in each row.

**Table 3:** Phenolic acids contents (pmol mg^–1^ dw), soluble free (SF), soluble ester-conjugated (SC), and cell wall-bound (CWB,) measured in seedlings of Chinese cabbage, white cabbage, and kale upon salt treatments (0–200 mM NaCl). Color gradation from light yellow to dark green comparatively represents values among species from the lowest to the highest. ND-not determined.

		Chinese Cabbage				White Cabbage				Kale			
		Treatments (NaCl mM)											
		0	50	100	200	0	50	100	200	0	50	100	200
**PA**	SF	9.45 ± 3.01a	9.97 ± 0.66a	10.02 ± 1.16a	5.35 ± 1.85b	18.33 ± 6.15a	11.78 ± 4.96a	11.46 ± 4.21a	11.70 ± 4.14a	2.61 ± 0.48a	2.08 ± 0.63a	2.47 ± 1.18a	2.58 ± 1.60a
	SC	69.68 ± 1.49ab	72.37 ± 7.17ab	74.61 ± 5.77a	63.20 ± 5.50b	155.46 ± 31.01a	149.93 ± 3.26a	148.82 ± 34.63a	143.58 ± 36.55a	134.31 ± 14.26a	129.72 ± 11.93a	128.16 ± 4.81a	125.49 ± 12.57a
	CWB	ND	ND	ND	ND	ND	ND	ND	ND	ND	ND	ND	ND
	Total	79.13 ± 4.17ab	82.34 ± 8.03a	84.6 3± 7.15a	68.55 ± 7.45b	173.79 ± 9.78a	161.71 ± 44.67a	160.28 ± 42.55a	155.28 ± 42.82a	136.92 ± 15.84a	131.81 ± 13.81a	130.63 ± 5.99a	128.07 ± 12.73a
**GaA**	SF	ND	ND	ND	ND	ND	ND	ND	ND	ND	ND	ND	ND
	SC	65.49 ± 9.26a	65.00 ± 10.34a	64.13 ± 11.11a	57.62 ± 9.65a	124.03 ± 11.81a	118.62 ± 9.89a	124.81 ± 9.28a	126.85 ± 15.24a	118.66 ± 6.65a	111.73 ± 19.38a	107.32 ± 7.30a	104.56 ± 13.73a
	CWB	ND	ND	ND	ND	ND	ND	ND	ND	ND	ND	ND	ND
	Total	65.49 ± 9.26a	65.00 ± 10.34a	64.13 ± 11.11a	57.62 ± 9.65a	124.03 ± 11.81 a	118.62 ± 9.89a	124.81 ± 9.28a	126.85 ± 15.24a	118.66 ± 6.65a	111.73 ± 19.38a	107.32 ± 7.30a	104.56 ± 13.73a
**pHBA**	SF	7.24 ± 3.31a	12.22 ± 3.29a	6.77 ± 1.42a	10.11 ± 4.35a	10.91 ± 2.98a	8.96 ± 2.11a	10.06 ± 0.95a	13.08 ± 5.76a	6.18 ± 1.67a	5.96 ± 0.34a	7.92 ± 3.53a	8.24 ± 3.17a
	SC	112.80 ± 23.13a	120.58 ± 6.49a	109.28 ± 7.96a	119.38 ± 11.29a	163.99 ± 11.73a	170.94 ± 14.42a	178.47 ± 15.51a	182.81 ± 16.37a	140.89 ± 6.28a	134.89 ± 3.32a	127.84 ± 5.79a	139.51 ± 11.58a
	CWB	ND	ND	ND	ND	ND	ND	ND	ND	ND	ND	ND	ND
	Total	120.03 ± 29.46a	132.80 ± 9.19a	116.05 ± 9.52a	129.50 ± 15.73a	174.89 ± 16.07a	179.90 ± 16.64a	188.54 ± 17.71a	195.89 ± 17.97a	147.07 ± 8.58a	140.85 ± 3.92a	135.76 ± 9.82a	147.75 ± 12.42a
**VA**	SF	ND	ND	ND	ND	ND	ND	ND	ND	ND	ND	ND	ND
	SC	291.96 ± 37.30a	263.46 ± 25.38a	248.10 ± 33.25a	305.44 ± 69.14a	142.38 ± 26.87a	126.06 ± 20.67a	135.85 ± 13.34a	149.09 ± 24.58a	153.14 ± 14.22a	155.80 ± 15.55a	141.84 ± 5.46a	146.49 ± 15.73a
	CWB	ND	ND	ND	ND	ND	ND	ND	ND	ND	ND	ND	ND
	Total	291.96 ± 37.30a	263.46 ± 25.38a	248.10 ± 33.25a	305.44 ± 69.14a	142.38 ± 26.87a	126.06 ± 20.67a	135.85 ± 13.34a	149.09 ± 24.58a	153.14 ± 14.22a	155.80 ± 15.55a	141.84 ± 5.46a	146.49 ± 15.73a
**CaA**	SF	ND	ND	ND	ND	ND	ND	ND	ND	ND	ND	ND	ND
	SC	34.19 ± 5.23a	26.98 ± 1.93b	25.76 ± 3.29b	21.62 ± 1.79b	51.30 ± 2.35a	47.37 ± 7.79a	47.55 ± 3.39a	47.34 ± 3.84a	149.61 ± 9.53a	141.03 ± 10.98ab	124.15 ± 10.93bc	114.07 ± 14.78c
	CWB	ND	ND	ND	ND	ND	ND	ND	ND	ND	ND	ND	ND
	Total	34.19 ± 5.23a	26.98 ± 1.93b	25.76 ± 3.29b	21.62 ± 1.79b	51.30 ± 2.35a	47.37 ± 7.79a	47.55 ± 3.39a	47.34 ± 3.84a	149.61 ± 9.53a	141.03 ± 10.98ab	124.15 ± 10.93bc	114.07 ± 14.78c
**SyA**	SF	ND	ND	ND	ND	ND	ND	ND	ND	ND	ND	ND	ND
	SC	72.60 ± 10.41a	68.48 ± 5.34a	69.23 ± 8.66a	72.08 ± 13.58a	98.61 ± 11.01a	98.98 ± 20.96a	97.46 ± 13.57a	97.63 ± 14.20a	51.13 ± 4.86a	51.13 ± 4.41a	51.18 ± 3.73a	53.28 ± 6.51a
	CWB	ND	ND	ND	ND	ND	ND	ND	ND	ND	ND	ND	ND
	Total	72.60 ± 10.41a	68.48 ± 5.34a	69.23 ± 8.66a	72.08 ± 13.58a	98.61 ± 11.01a	98.98 ± 20.96a	97.46 ± 13.57a	97.63 ± 14.20a	51.13 ± 4.86a	51.13 ± 4.41a	51.18 ± 3.73a	53.28 ± 6.51a
**pCoA**	SF	4.90 ± 2.92ab	6.20 ± 1.19a	2.46 ± 0.70b	3.12 ± 0.95ab	8.59 ± 3.24a	5.43 ± 1.37a	6.09 ± 1.63a	5.30 ± 2.27a	5.68 ± 2.41a	3.11 ± 0.82a	2.94 ± 1.48a	3.23 ± 2.15a
	SC	116.74 ± 30.51a	86.67 ± 15.09ab	86.17 ± 20.10ab	73.20 ± 10.40b	147.45 ± 32.86a	161.25 ± 17.85a	163.84 ± 22.88a	173.81 ± 38.67a	177.90 ± 20.78a	119.80 ± 19.58b	110.65 ± 12.85bc	81.52 ± 8.01c
	CWB	6.20 ± 3.11a	4.85 ± 1.70a	3.23 ± 0.64a	3.47 ± 0.74a	22.14 ± 8.95a	23.03 ± 7.08a	23.33 ± 6.42a	26.46 ± 11.17a	6.64 ± 2.35a	4.82 ± 0.89a	5.14 ± 1.39a	6.10 ± 1.38a
	Total	127.84 ± 34.47a	97.72 ± 15.96ab	91.86 ± 23.06ab	79.79 ± 11.04b	178.18 ± 47.41a	189.70 ± 22.79a	193.26 ± 28.73a	205.57 ± 45.73a	189.08 ± 23.25a	127.73 ± 23.28b	118.15 ± 14.22bc	90.85 ± 8.97c
**SiA**	SF	180.29 ± 42.77ab	302.02 ± 100.08a	153.78 ± 36.94b	261.04 ± 101.60ab	162.06 ± 59.92a	102.26 ± 36.36a	147.12 ± 44.28a	111.45 ± 28.05a	115.01 ± 93.67a	81.62 ± 14.93a	178.18 ± 236.05a	335.75 ± 221.43a
	SC	4348.0 ± 334.00a	4468.30 ± 112.11a	4655.07 ± 674.13a	4524.51 ± 522.53a	5008.74 ± 613.86a	5504.28 ± 1058.00a	5562.21 ± 1278.82a	5578.33 ± 1004.63a	6475.78 ± 575.80a	6511.83 ± 485.33a	6289.38 ± 260.04a	6492.59 ± 681.11a
	CWB	306.55 ± 78.89a	295.63 ± 107.36a	257.14 ± 54.39a	263.01 ± 84.34a	636.51 ± 90.56a	678.64 ± 119.39a	666.02 ± 119.67a	613.25 ± 128.86a	483.09 ± 72.58a	449.33 ± 77.03a	427.39 ± 57.79a	402.40 ± 91.84a
	Total	4834.8 ± 290.75a	5065.95 ± 163.74a	5065.99 ± 728.55a	5048.56 ± 468.03a	5807.30 ± 677.05a	6285.18 ± 1136.82a	6375.35 ± 1373.16a	6303.03 ± 1123.34a	7073.88 ± 669.84a	7042.78 ± 588.29a	6894.94 ± 117.30a	7230.73 ± 620.73a
**FA**	SF	20.15 ± 5.92a	18.12 ± 1.96a	15.99 ± 4.05a	18.33 ± 3.53a	7.12 ± 4.00a	4.54 ± 1.41a	5.82 ± 1.32a	4.47 ± 2.32a	1.46 ± 1.07b	1.32 ± 0.71b	1.47 ± 0.75b	6.41 ± 3.38a
	SC	204.62 ± 10.90a	211.87 ± 5.40a	205.38 ± 29.09a	187.82 ± 18.77a	365.77 ± 58.68a	368.83 ± 77.60a	368.94 ± 63.52a	378.25 ± 84.14a	253.05 ± 22.47a	237.43 ± 20.28a	240.84 ± 15.96a	261.17 ± 11.06a
	CWB	28.25 ± 4.52a	29.48 ± 8.75a	23.96 ± 3.36a	24.19 ± 3.25a	99.14 ± 34.37a	79.05 ± 27.59a	77.82 ± 25.64a	89.62 ± 27.38a	30.04 ± 8.20a	26.04 ± 3.46a	31.34 ± 5.69a	34.94 ± 8.60a
	Total	253.02 ± 14.59a	259.47 ± 9.93a	245.33 ± 35.83a	230.34 ± 21.64a	469.18 ± 95.30a	451.51 ± 108.24a	451.42 ± 93.64a	471.44 ± 118.46a	284.55 ± 31.80a	264.80 ± 24.00a	273.66 ± 22.65a	302.53 ± 17.81a
**SA**	SF	80.23 ± 21.57a	70.81 ± 21.17a	62.43 ± 13.86a	25.97 ± 2.08b	47.00 ± 11.33 a	40.48 ± 4.90a	36.59 ± 10.05a	32.22 ± 9.40a	27.08 ± 3.36a	25.83 ± 6.41ab	20.02 ± 0.73bc	15.73 ± 2.01c
	SC	95.39 ± 25.08a	77.29 ± 22.68a	71.87 ± 9.72a	36.48 ± 3.47b	82.89 ± 14.84a	79.94 ± 16.82a	65.14 ± 8.00a	58.75 ± 11.27a	54.71 ± 3.44a	50.22 ± 6.18a	49.57 ± 6.17a	43.47 ± 7.21a
	CWB	ND	ND	ND	ND	ND	ND	ND	ND	ND	ND	ND	ND
	Total	175.62 ± 46.53a	148.10 ± 42.60a	134.30 ± 20.91a	62.45 ± 4.49b	120.49 ± 24.20a	120.42 ± 24.12a	101.73 ± 18.12a	90.97 ± 22.07a	81.78 ± 4.17a	76.05 ± 10.45a	69.58 ± 6.77ab	59.21 ± 8.69b

Data labeled with different letters (a–c) differ significantly for each species, control and treatments, at *p* < 0.05.

## Supplementary Materials

The following are available online at https://www.mdpi.com/2223-7747/8/6/155/s1, Figure S1: Representative UPLC–MS/MS chromatograms (A–J) of conjugated phenolic acids in a kale (*Brassica oleracea* var. *acephala*) extract: salicylic acid (1), ferulic acid (2), sinapic acid (3), 4-coumaric acid (4), syringic acid (5), caffeic acid (6), vanillic acid (7), 4-hydroxybenzoic acid (8), gallic acid (9), protocatechuic acid (10). Table S1: Specific metabolites in *Brassica* seedlings upon salinity stress (0–200 mM NaCl). Table S2: Correlation matrix (Pearson (n)). Table S3: Eigenvalues. Table S4: Factor loadings and correlations between variables and factors. Table S5: Squared cosines of the variables. Table S6: Squared cosines of the observations.
