# Supplementary material for: Involvement of Phenolic Acids in Short-Term Adaptation to Salinity Stress is Species-Specific among Brassicaceae
Source: Plants (Basel). 2019 Jun 6;8(6):155. doi: 10.3390/plants8060155 (PMC6631191; doi:10.3390/plants8060155)
Supplement: Supplementary file 1 [file plants-08-00155-s001.zip › Supplemental files_Salopek-Sondi/Supplemental fig1..pdf]

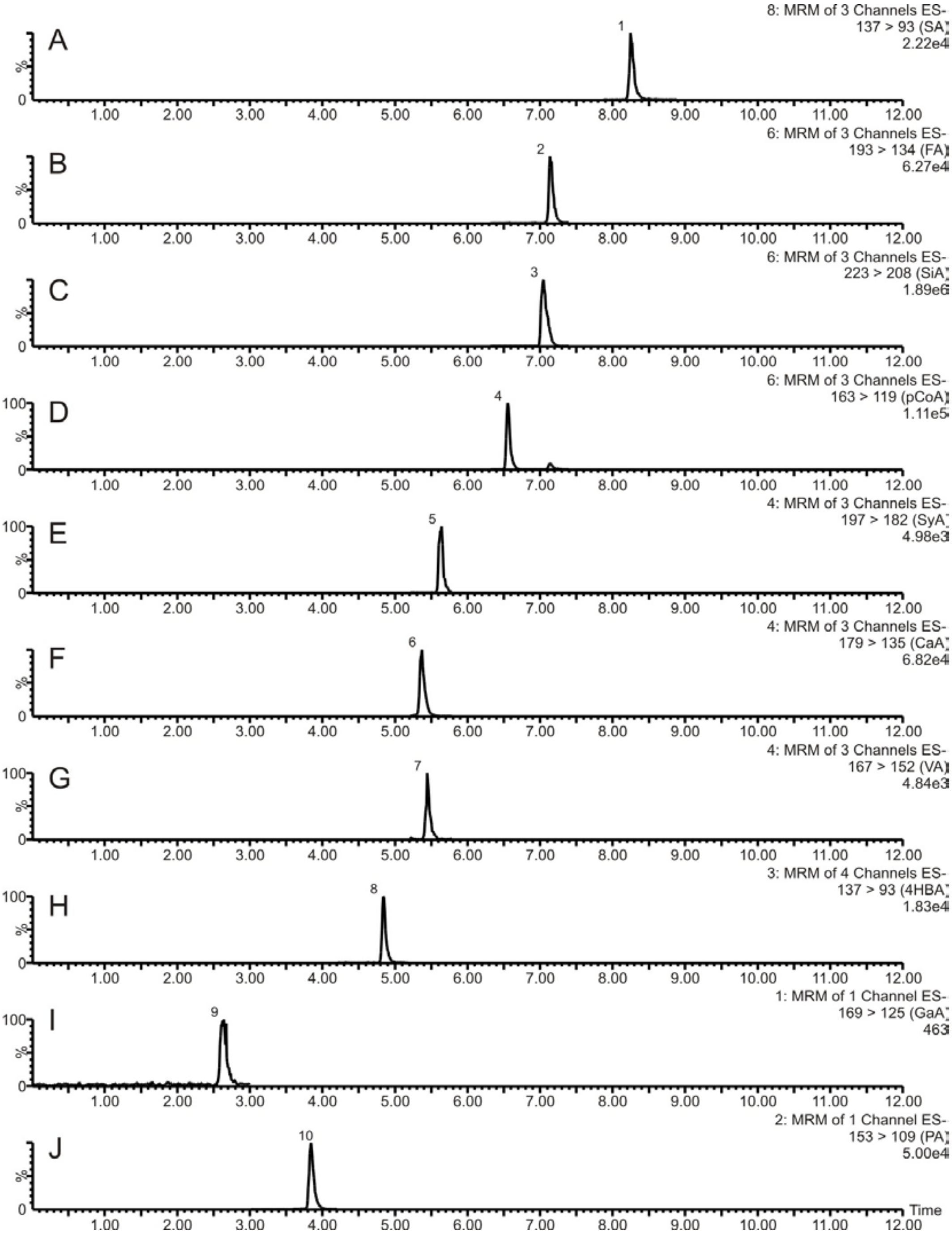

Supplemental Figure S1. Representative UPLC-MS/MS chromatograms (A-J) of conjugated phenolic acids in a kale (*Brassica oleracea* var. *acephala*) extract: salicylic acid (1), ferulic acid (2), sinapic acid (3), 4-coumaric acid (4), syringic acid (5), caffeic acid (6), vanillic acid (7), 4-hydroxybenzoic acid (8), gallic acid (9), protocatechuic acid (10).
